# Antifungal Activity of Plasmacytoid Dendritic Cells and the Impact of Chronic HIV Infection

**DOI:** 10.3389/fimmu.2017.01705

**Published:** 2017-12-04

**Authors:** Samuel Maldonado, Patricia Fitzgerald-Bocarsly

**Affiliations:** ^1^Rutgers School of Graduate Studies, Newark, NJ, United States; ^2^Department of Pathology and Laboratory Medicine, New Jersey Medical School, Newark, NJ, United States

**Keywords:** plasmacytoid dendritic cells, C-type lectin receptors, toll-like receptors, innate antifungal immunity, HIV

## Abstract

Due to the effectiveness of combined antiretroviral therapy, people living with HIV can control viral replication and live longer lifespans than ever. However, HIV-positive individuals still face challenges to their health and well-being, including dysregulation of the immune system resulting from years of chronic immune activation, as well as opportunistic infections from pathogenic fungi. This review focuses on one of the key players in HIV immunology, the plasmacytoid dendritic cell (pDC), which links the innate and adaptive immune response and is notable for being the body’s most potent producer of type-I interferons (IFNs). During chronic HIV infection, the pDC compartment is greatly dysregulated, experiencing a substantial depletion in number and compromise in function. This immune dysregulation may leave patients further susceptible to opportunistic infections. This is especially important when considering a new role for pDCs currently emerging in the literature: in addition to their role in antiviral immunity, recent studies suggest that pDCs also play an important role in antifungal immunity. Supporting this new role, pDCs express C-type lectin receptors including dectin-1, dectin-2, dectin-3, and mannose receptor, and toll-like receptors-4 and -9 that are involved in recognition, signaling, and response to a wide variety of fungal pathogens, including *Aspergillus fumigatus, Cryptococcus neoformans, Candida albicans*, and *Pneumocystis jirovecii*. Accordingly, pDCs have been demonstrated to recognize and respond to certain pathogenic fungi, measured *via* activation, cytokine production, and fungistatic activity *in vitro*, while *in vivo* mouse models indicated a strikingly vital role for pDCs in survival against pulmonary *Aspergillus* challenge. Here, we discuss the role of the pDC compartment and the dysregulation it undergoes during chronic HIV infection, as well as what is known so far about the role and mechanisms of pDC antifungal activity.

## Introduction

An estimated 33 million people were living with HIV/AIDS worldwide in 2015 (1). Although the development of combined antiretroviral therapy has allowed HIV patients to suppress viral replication and live longer lifespans (2, 3), HIV evades the immune system and persists, causing chronic immune activation and inflammation (4). The persistence of infection and immune activation leads to significant detrimental changes that occur in the body, and pose major threats to patients’ well-being; in fact, in 2010, HIV/AIDS was still the fifth leading cause of disease burden worldwide (5).

Arguably the most significant pathological process faced by treated and untreated patients alike is the erosion of the immune system. HIV infection famously depletes CD4^+^ T-helper (T_H_) cells, first quickly in the acute phase of infection, followed by a near-total recovery in numbers, and finally a steady decline over a number of years (4) leaving the adaptive immune system at a tremendous disadvantage. T-cell depletion occurs *via* mechanisms such as direct infection, bystander effects due to chronic inflammation, and senescence (4, 6). The dysfunction of the immune system is also much broader; almost every known immune cell type has been associated with a dysfunction due to chronic HIV infection. Because HIV gains access to a cell *via* its interaction with CD4 and co-receptors CXCR4 and CCR5, it also has the capacity to infect other cells that express those receptors, including monocytes, macrophages, and plasmacytoid dendritic cells (pDCs) (4). In addition, the immune system may be further dysregulated due to chronic innate immune activation and the continued production of normally beneficial cytokines (7).

This erosion of the immune system leaves patients hyper-susceptible to a variety of opportunistic infections, including fungal infections not commonly seen in the general population. For example, *Candida albicans* infection is the most common fungal infection in HIV patients. While it usually presents as oropharyngeal thrush, it can span a broad spectrum of severity from asymptomatic to invasive candidiasis (8). The most common systemic fungal infection in the HIV population is cryptococcal meningitis, caused by *Cryptococcus neoformans*. Cryptococcal meningitis causes 181,000 deaths a year in HIV-positive patients, accounting for 15% of global AIDS-related deaths (9, 10). *Aspergillus fumigatus* is the causative agent of invasive Aspergillosis, which is less common in the context of HIV infection but is particularly aggressive and difficult to treat, resulting in a median survival of only 3 months after diagnosis (11, 12). These and other pathogenic fungi take advantage of an HIV patient’s impaired immune system. Studies early in the HIV epidemic suggested that HIV patients are much more likely to develop opportunistic infections when two events occur: absolute CD4^+^ cell counts drop below 250 cells/mm^3^, and interferon (IFN)-α production by virus-stimulated peripheral blood mononuclear cells (PBMCs) drops below 300 IU/ml (13). It is the latter that makes pDCs a cell type of great interest when studying HIV infection, as they are the body’s most potent producers of type-I IFNs. pDCs produce up to 100-fold more IFN-α than any other cell type in response to viral stimulation and serve as an important link between the innate and the adaptive branches of the immune system (14–16). pDC IFN production and influence over the rest of the immune response has made these cells the subject of extensive investigation in human and mouse models. Importantly, murine systems do not perfectly represent human ones, and studies on mouse pDC function must also be investigated in humans. For example, toll-like receptor (TLR) 9 is broadly expressed by murine myeloid cells, but is more limited to pDCs and B-cells in humans (17). Similarly, murine pDCs commonly produce IL-12 while human pDCs rarely, if ever, produce IL-12 (18).

Plasmacytoid dendritic cell dysregulation during chronic HIV infection has garnered attention due to the potentially far-reaching effects on patient outcome and well-being. However, an interesting development in the field of pDC research is the investigation into their role in fungal infection. Studies have demonstrated that pDCs have the machinery needed to recognize and respond to fungal stimulation, that they in fact do respond with certain cellular functions, and that they are necessary *in vivo* for a successful antifungal immune response. In this review, we outline the current paradigm concerning pDCs’ role in viral immunity and describe the dysregulation of the pDC compartment during chronic HIV infection. We then switch gears to the role of pDCs in antifungal immunity, where we highlight the components of fungal immunity that pDCs possess, and summarize what has been discovered to date concerning the mechanisms of pDC antifungal activity.

## pDCs in Antiviral Immunity

Both the innate and the adaptive immune responses play active roles in combating viral infections such as HIV. The adaptive immune response includes a strong CD4^+^ and CD8^+^ T-cell response, as well as the production of neutralizing antibodies that begins during the acute phase of infection (19, 20). Mobilization of the adaptive immune response depends in part on pDCs, a lineage-negative subtype of dendritic cells that derives its name from a plasma-cell-like morphology. Although pDCs comprise only 0.2–0.8% of PBMC (14, 15, 21), their powerful immunomodulating capabilities make pDCs highly influential to the development of the immune response to HIV infection, and a lack of sufficient pDC numbers or function corresponds with high viral loads and opportunistic infections (22).

Human pDCs are generally found in peripheral blood and lymphoid tissue, so if an infection occurs in a different tissue (e.g., the lung or mucosa), they must migrate to sites of infection or inflammation by responding to chemokines. This occurs primarily *via* the chemokine receptors CMKLR1, CCR7, CXCR3, and CXCR4 (22–24) as well as through homing molecules, such as CD62L, which binds L-selectin and allows pDCs to track to high endothelial venules and the gut homing receptor α4β7 (25). Once at the site of infection, pDCs directly recognize and respond to viral stimulation. The initial interaction between pDCs and HIV virions is possible because pDCs express the surface molecules that are targeted by HIV: CD4, CCR4, and CXCR5, as reviewed in Ref. (16). The interaction between the virus and CD4 allows the pDC to endocytose the virus, which induces activation in a TLR-dependent or -independent manner.

In the TLR-dependent pathway, the endocytosed virus is delivered to endosomes in pDC that contain TLR-7 and 9. Acidification of the endosomes allows TLR-7 and 9 to access viral RNA or unmethylated CpG motifs, including that in viral DNA, respectively (26, 27). These TLRs have the capacity to recognize a host of viral, bacterial, and fungal nucleic acids, as well as small molecule artificial ligands such as imiquimod and resiquimod (28, 29). Ligation of TLR-7 or 9 induces a MyD88-dependent activation pathway that ultimately leads to pDC activation and cytokine production (30). Although less well-characterized than the TLR-dependent pathway, evidence exists for a TLR-independent activation pathway in pDCs. For example, one study found that IFN-α production by murine pDCs in response to Sendai virus was not abrogated in MyD88^−/−^ mice and was not dependent on endosomal acidification, both of which are required for TLR9 signaling (31). Another study found that murine pDC IFN-α production in response to HSV-1 was not completely dependent on TLR9 (32). However, specific mechanisms of TLR-independent pDC activation and cytokine production are still under investigation.

Both the TLR-dependent and -independent signaling pathways converge on interferon regulatory factor (IRF) 7, the master regulator of IFN production which is constitutively expressed by pDCs at uniquely high levels (30, 33, 34). Phosphorylation of IRF7 causes it to translocate into the nucleus and induce the transcription of IFN genes, producing high concentrations of type-I IFN (35). TLR-7 and -9 ligation also lead to the activation of transcription factor NF-κB, which induces the production of inflammatory cytokines/chemokines and the upregulation of co-stimulatory molecules (21, 36). Activated pDCs can then produce type-I IFNs, type-III IFNs, and a network of other cytokines and chemokines. They also have the ability to become antigen-presenting cells, at which point they develop a morphology resembling conventional DCs (cDCs) (36–38).

Perhaps the most significant contribution of pDCs to antiviral immunity is the production of massive amounts of type-I IFNs (mainly α and β) in response to viral stimulation. Most notably, pDCs can produce 100–1,000 times more type-I IFN than any other cell type (39–41). It has even been reported that as much as 60% of genes expressed in an activated pDC are dedicated to the production of type-I and type-III IFNs (42). Type-I IFNs all bind the same receptor, the IFN-α/β receptor, which is widely expressed by most nucleated cells in the body. The IFN-α/β receptor is composed of two subunits, IFNAR1 and IFNAR2, that form a heterodimer upon ligation of type-I IFN (43). IFNAR-associated Janus kinases then phosphorylate STAT1 and STAT2, which form a complex with IRF9 called the ISGF3 complex. The resulting signaling cascade activates gene transcription for factors that produce an antiviral state in surrounding tissues and mobilizes virtually all types of immune cells to combat viral infection (44, 45). For example, IFN-α acts as a survival, maturation, and cytokine/chemokine production factor for pDCs (46, 47) and cDCs (48), activates natural killer cells (26), biases the immune system toward a T_H_1 response (49, 50), primes CD8^+^ T-cells and induces memory CD8^+^ T-cells (51–53), promotes the development of regulatory T (Treg) cells (54), and enhances antibody production against soluble antigen (55). These effects, among others, are critical for the inhibition of viral replication and are why type-I IFNs are arguably the most important cytokines in antiviral immunity.

In addition to type-I IFNs, pDCs also produce type-III IFNs (IFN-λ1–3) after viral stimulation (38). The IFN-λ receptor is expressed on a much more limited scale than the IFN-α/β receptor—primarily epithelial cells and pDCs (38, 56)—suggesting that the biological activity of IFN-λ is more narrow than that of type-I IFN. However, type-I and type-III IFN share many common features: their signaling pathways include many of the same factors, including STAT1, STAT2, and the formation of the ISGF3 complex; they also activate several common genes (57, 58). Functionally, IFN-λ also has antiviral effects, especially for the protection of epithelial cells (59), the enhancement of TLR-mediated signaling in pDCs (56), development of Treg cells (60), and biasing toward a T_H_1 response by reducing IL-13 expression (61). However, it has also been reported that IFN-λ can be antagonistic to type-I IFN antiviral activity (62).

Other than IFNs, pDCs are able to produce several other cytokines and chemokines. NF-κB activation after viral stimulation induces pDCs to produce inflammatory cytokines and chemokines such as IL-12 (in murine but not human pDC), TNF-α, RANTES, IL-10, IP-10, IL-6, MIP-1α, and MIP-1β (36, 63). One study identified 12 chemokines that are produced in three distinct waves by pDCs in the hours following influenza virus infection (64). Furthermore, pDCs are also capable of other innate immune functions. Although resting pDCs are less efficient than myeloid DCs at capturing, processing, and presenting antigen (65, 66), activation of pDCs in response to influenza virus induces a change into a dendritic morphology and the upregulation of MHC-II, enhancing their ability to present antigen to naïve and memory T-cells (67, 68). In 2012, Tel et al. reported that pDCs could not only present tumor antigens but could also directly kill tumor cells. They termed these cells “killer pDCs” (69). Another interesting feature of the pDC is the expression of several surface receptors usually restricted to myeloid cells. On pDCs, these receptors include the C-type lectin receptors (CLRs) dectin-1, dectin-2, dectin-3, and mannose receptor (MR) (70–72), which will be discussed later. These receptors are of particular interest because they are involved in antifungal immunity.

## pDC Dysregulation During Chronic HIV Infection

Despite the impressive antiviral functions of pDCs, HIV still manages to evade the immune system and persist in the body. The observation that HIV patient blood cells produced less IFN after stimulation led to the discovery of what were termed natural IFN-producing cells, which would later be known as pDCs (73–76). After the discovery of pDCs as the major IFN-α-producing cell, observations surfaced that showed chronic HIV infection leads to a decline in pDC number and function (77). The decline in absolute pDC numbers correlated directly with a decline in CD4^+^ T-cell count and increased viral load (22, 78), while pDC functional decline (percent of IFN-α-producing pDC after viral stimulation) correlated with an increase in patient viral load, but not CD4^+^ T-cell count (76). Although ART treatment results in a partial recovery of pDC numbers, they do not make a full recovery (79). The specific cause of this decline in pDC number is still under investigation. Studies on macaque models indicate that pDCs are recruited to lymph nodes where they die rapidly (80, 81); this was supported by a study that found higher numbers and accelerated pDC death in human lymph nodes (82). Other studies show that pDCs are susceptible to HIV infection, which can cause syncytia formation and cell death (83, 84). Other possible mechanisms include recruitment to mucosal tissues, bystander apoptosis similar to T-cells, and bone marrow suppression (85).

Along with lower numbers, HIV also causes functional differences in pDCs. One of the earliest observations in HIV patients was the high levels of circulating IFN-α seen in some untreated patients, which was associated with disease progression (13, 86). The pDCs that accumulate in HIV patient lymph nodes were also described as producing significantly higher levels of IFN-α than non-infected healthy controls (82). This suggests that at certain points during infection, pDCs are chronically activated and are continuously producing IFN-α. This chronic immune activation may be driving pathology, including the immune exhaustion of T-cell compartments (7, 87). Supporting this hypothesis, one study found that humanized pDC-depleted mice infected with HIV-1 show dramatically reduced cell death and immune cell depletion, a phenomenon they suggested stems from the lack of persistent pDC activation and IFN production (88). In addition, other pDC functions are found to be dysregulated in HIV patients: pDCs become less able to stimulate T-cell proliferation in mixed lymphocyte reactions (89), show a partial activation phenotype (90), and have a reduced ability to respond to TLR7 and -9 agonists (91).

Another area of research concerns the possibility that pDCs may be inducing the differentiation of human Treg cells that lead to tolerogenicity and suppression of T-cell proliferation during chronic HIV infection. Human pDCs have been shown to induce both CD8^+^ and CD4^+^ Treg cell development in certain circumstances. For example, pDCs stimulated with type B CpG oligodeoxynucleotides (CpGb) induced CD4^+^CD25^−^ naïve T-cells to differentiate into CD4^+^CD25^+^ Treg cells, which suppressed T-cell proliferation and promoted tolerance (92). Another study found that pDCs activated with CD40L induced naïve CD8^+^ T-cells to proliferate, but the resulting CD8^+^ T-cells were poorly cytotoxic, had poor responses to secondary re-stimulation, and produced IL-10 (93). To that end, a study by Boasso et al. (94) found that T-cell proliferation in HIV-infected patients was being inhibited by pDCs through the expression of indoleamine-2,3-dioxygenase (IDO) (94). IDO is the first and rate-limiting enzyme in the catabolism of tryptophan, and its expression can be used by APCs as an immune modulator to limit T-cell proliferation (95). In the study, IDO mRNA expression in the blood of HIV-infected patients was directly correlated with viral load, and was responsible for impaired CD4^+^ T-cell proliferation. Using flow cytometric analysis, they found that pDCs were the main producers of IDO, and that pDC IDO expression was a direct response to HIV that did not require the presence of other leukocytes. This phenomenon may represent yet another way that pDCs contribute to the dysfunction of the immune system during chronic HIV infection. In addition, another study found that IDO expression by pDCs blocks T-cell differentiation into T_H_17 cells, which could further negatively impact adaptive immunity to fungal infection (96). This dysfunction, along with the dysregulation of virtually every immune cell type, leaves patients at higher risk of opportunistic infections and other comorbidities, including fungal infections with *C. albicans* and *C. neoformans*.

## Select Components of the Immune Response to Fungal Infection

A discussion of HIV patient susceptibility to fungal infections requires an understanding of normal antifungal immunity. Although some fungi are commensal with the human host, this review focuses on pathogenic fungal infections. Fungal pathogens, such as *A. fumigatus* and *C. neoformans*, tend to be ubiquitous to the environment and are introduced into the human host through inhalation of spores or small yeast cells (97, 98). In the case of *A. fumigatus* and other sporulating fungi, spores (termed “resting conidia,” RC) that reach a suitable host begin to swell (termed “swollen conidia,” SC), and germinate (termed “germinating conidia,” GC), finally growing into filamentous hyphae (99). Fortunately, fungal pathogens express pathogen-associated molecular patterns (PAMPs), which are not found on mammalian cells and alert the immune system to the presence of non-self organisms (100). PAMPs can vary depending on the species, growth stage, and environment of the fungus (101, 102). Three significant PAMPs on fungal cell walls include β-glucans (polymers of glucose, typically with 1,3 linkages and occasional 1,6 branches), chitin (a polymer of *N*-acetylglucosamine), and mannans (polymers of mannose linked to fungal proteins) (97, 103). Fungi that make it into a human host can be recognized by innate immune cells through pattern recognition receptors (PRRs), such as TLRs and CLRs (100).

Once the PAMPs on fungi are recognized by cellular PRRs, a complex immune response begins that, incorporates both the innate and adaptive branches of the immune system (104). Phagocytes, such as monocytes, neutrophils, and macrophages, phagocytose and kill fungal pathogens directly (105), spread out over hyphae and kill them *via* oxidative and non-oxidative means, and in the case of neutrophils produce extracellular traps (106, 107). In addition to uptake and direct killing, cDCs also transport and present fungal antigen to T-cells and induce their differentiation into T_H_1, T_H_17, or Treg cells, depending on the environment and the fungus (108–110). While T_H_1 cells are thought to be the most protective, patients with defects in the T_H_1 compartment are not overly susceptible to certain fungal infections (97). This is because the T_H_17 compartment is also protective and sometimes essential to fungal clearance, depending on the species and route of infection (111). Conversely, inborn errors in IL-17 immunity can result in chronic mucocutaneous candidiasis in humans (112), and more broad susceptibility to *C. albicans* infection in mice, including cutaneous and oropharyngeal infection (113). Finally, the development of protective antibodies after vaccination of mice with *C. albicans* adhesins suggests that humoral immunity may also get involved during fungal infection, although in those studies the main effector cells responsible for vaccine efficacy were CD4+ T-cells, and neither B-cell transfer nor passive immunization with serum of vaccinated mice were protective to unvaccinated mice (114).

The TLRs involved in fungal recognition that are expressed on pDCs are TLR4 and TLR9 (17, 35). TLR4 (CD284) is mainly known for recognizing bacterial lipopolysaccharide, but it also appears to recognize mannan structures of fungal cell walls, leading to cytokine/chemokine production and recruitment of neutrophils. TLR4 is composed of a 608 residue extracellular domain, which contains 21 leucine-rich repeats and can be subdivided into N-, central, and C-terminal domains, and a 187-residue intracellular domain that signals *via* the TLR-associated adaptor MyD88 (115). Interruption of TLR4 signaling has been shown to increase susceptibility to *C. albicans* (116), *A. fumigatus* (117, 118), and *Pneumocystis jirovecii* (119) in mice. However, it is worth noting that TLR4 signaling did not induce cytokine production after recognizing mannans on *C. neoformans* cell walls (120), indicating fungal species-specific responses.

TLR9 (CD289) recognizes unmethylated CpG DNA sequences, which may allow it to recognize fungal DNA. As mentioned earlier, TLR9 is located in endosomes of pDCs, where it is known for responding to viral DNA in a MyD88-dependent manner. TLR9 has 25 intra-endosomal leucine-rich repeats, but a detailed structure for TLR9 has not yet been discovered (121). Studies show TLR9 induces IL-10 production by macrophages in response to *C. albicans* (120), pro-inflammatory cytokine secretion by mouse bone marrow-derived dendritic cells in response to *A. fumigatus* DNA (122), and IL-12p40 production and CD40 upregulation in DCs in response to *C. neoformans* (123). It is interesting to note that while MyD88 serves as a signaling adaptor for all of the TLRs except TLR3, patients with MyD88 deficiencies or mutations do not suffer from severe or recurrent fungal infections, suggesting that TLR signaling enhances but is not indispensable for antifungal immune responses (124).

C-type lectin receptors are a large family of receptors that contain C-type lectin-like domains (CTLDs), which, depending on the receptor, recognize a wide array of carbohydrates, proteins, or lipids (125). CLRs are a diverse family of receptors with a multitude of functions, but this review will focus on the four CLRs that have a role in fungal recognition and have been identified on human pDCs: dectin-1, dectin-2, dectin-3, and MR.

Dectin-1 (CLEC7A) recognizes β(1,3)- and β(1,6)-linked glucans, which are present on bacterial and fungal cell walls. B-glucans can comprise up to 50% of the cell walls of common pathogenic fungi, including *A. fumigatus, C. neoformans, P. jirovecii*, and *C. albicans* (126–130). Dectin-1 is composed of an extracellular CTLD, a single transmembrane domain, and an intracellular immunoreceptor tyrosine-based activation motif (ITAM) (126). The dimerization of dectin-1 and the phosphorylation of its intracellular ITAM recruits spleen tyrosine kinase (Syk), and induces a signaling cascade that includes the signaling adaptor caspase recruitment domain-containing protein 9 (CARD9). CARD9 is a major point of regulation within the signaling cascade of several CLRs, including dectin-1 and dectin-2, and is indispensable for the cell activation and cytokine production caused by dectin-1 and -2 ligation (131, 132). CARD9 deficiency has been linked to chronic and recurring fungal infections, especially by *C. albicans* (133, 134). In addition, pDCs from CARD9 knockout mice produced significantly less IL-6 and TNF-α than wild-type mice in response to influenza virus, while type-I IFN production was unaffected (135). Dectin-1/Syk/CARD9 signaling ultimately leads to the canonical activation and nuclear translocation of NF-κB subunits Rel-A (p65) and c-Rel. Dectin-1 also activates the NF-κB unit Rel-B through a non-canonical pathway, *via* Raf-1 instead of Syk (136). Dectin-1 has been shown to have an array of immune functions, including the recognition, uptake, and killing of fungi (137, 138), as well as cellular signaling leading to maturation, production of a respiratory burst, cytokine and chemokine production, enhanced survival and differentiation of T_H_ cells that helps control fungal infection in the GI tract, and cytotoxic T-lymphocyte priming (128, 139–143).

Dectin-2 (CLEC6A) recognizes α-mannose structures present on the surface of many viruses, bacteria, and fungal pathogens. Dectin-2 has been shown to have a role in immunity to fungi, including *C. albicans, A. fumigatus*, and non-encapsulated *C. neoformans*, with a preference for hyphal as opposed to yeast or conidial forms of fungal cell walls (144–146). It is composed of an extracellular CTLD and a short cytoplasmic tail, which couples with FcRγ to initiate downstream signaling. The phosphorylation of FcRγ again recruits Syk and CARD9 (147, 148). Dectin-2 signaling also induces the activation of NF-κB, but in contrast to dectin-1, dectin-2 selectively activates the c-Rel subunit of NF-κB *via* MALT1. This selectivity suggests that dectin-2 is more specific for the production of cytokines that induce T_H_17 polarization (149). Dectin-2 signaling induces internalization of the fungus and the production of an array of cytokines, including TNF-α and IL-1β, 2, 6, 10, 12, and 23 (145, 147). Furthermore, blocking dectin-2 in mice abrogated the development of T_H_1 and T_H_17 cells, which left mice significantly more susceptible to *C. albicans* infection (150).

Similar to dectin-2, dectin-3 (CLEC4D, CLECSF8) also has a single extracellular CRD with a short cytoplasmic tail. The CRD of dectin-3 is highly homologous with that of dectin-2, and similarly recognizes α-mannans (151). In fact, dectin-3 was found to dimerize with dectin-2, and the resulting heterodimer was more adept at recognizing fungal PAMPs than the respective homodimers (152). Although the cytoplasmic tail of dectin-3 lacks a signaling motif and does not associate with any known signaling adaptor, including FcRγ, it does induce signaling *via* Syk and the CARD9/Bcl10/Malt1 complex and activates NF-κB (153). The cellular processes that are induced include phagocytosis, cytokine production, and a respiratory burst in macrophages (154). To illustrate its physiological relevance, murine bone marrow-derived macrophages pretreated with dectin-3-blocking monoclonal antibodies were inhibited from NF-κB nuclear translocation and cytokine production after *C. albicans* stimulation, and mice treated with dectin-3-blocking antibody succumbed rapidly to sub-lethal *C. albicans* IV challenge (152). Similarly, mice deficient in dectin-3 were more susceptible to DSS-induced colitis, and murine bone marrow-derived macrophages were impaired in phagocytic and fungicidal ability, NF-κB nuclear translocation, and cytokine production (155). Conversely, no role was found for dectin-3 in defense against *C. neoformans*, as dectin-3-deficient mice did not differ in pulmonary leukocyte recruitment, pulmonary cytokine profiles, or overall survival after pulmonary *C. neoformans* challenge. In addition, macrophages and DCs isolated from dectin-3 knockout mice did not differ in their phagocytic or fungicidal ability against *C. neoformans* (156).

Mannose receptor (CD206) is a highly endocytic receptor with three categories of extracellular binding domains, a single transmembrane domain, and a short cytoplasmic tail (157). The extracellular binding domains include an N-terminal cysteine-rich (CR) domain, which binds sulfated carbohydrates; a fibronectin type II (FNII) domain, which binds collagen; and eight CTLDs, which bind carbohydrates that terminate in d-mannose, l-fucose, or *N*-acetyl glucosamine, as reviewed in Ref. (158, 159). While the CR and FNII extracellular domains are involved in glycoprotein homeostasis (160, 161), it is the CTLDs that are responsible for recognizing carbohydrate entities on pathogens (159). For one particularly interesting example, MR binds the gp120 glycoprotein on HIV and mediates binding of the virus by dendritic cells (70) and macrophages (162). In the context of fungal infection, the MR has been shown to enhance uptake and clearance of pathogenic fungi, including *A. fumigatus* (163) *C. albicans* (164, 165), *C. neoformans* (166), and *Pneumocystis carinii* (167). However, MR seems to not be phagocytic in its own right; MR was unable to induce phagocytosis of its ligands when transfected onto Chinese hamster ovary cells (168). MR instead influences the signaling of other receptors. For example, Tachado et al. (169) discovered that human embryonic kidney 293 cells required transfection of both TLR2 and the MR in order to produce IL-8 in response to *Pneumocystis*. Similarly, in alveolar macrophages, *Pneumocystis* challenge led to direct interaction of TLR2 and MR, while blocking MR and TLR2 simultaneously abrogated IL-8 production (169). The downstream results of MR signaling are varied and dependent on many factors, including signaling from other receptors and the route of ligand delivery (158). A summary of the TLRs and CLRs discussed here can be found in Table 1.

**Table 1:** A summary of the pattern recognition receptors (PRRs) on plasmacytoid dendritic cells (pDCs) that have a role in antifungal immunity.

Receptor	Structure	Fungal ligand	Cellular functions	Fungal pathogen	Reference
TLR4 (CD284)	Extracellular 608 residue domain, composed of an N-, central, and C-terminal domain. 21 leucine-rich repeats	Mannan structures	Cytokine/chemokine production, recruitment of neutrophils	*Candida albicans, Aspergillus fumigatus, Pneumocystis*. Not TLR4-dependent in *C. neoformans* models.	(17, 35, 115–120)
TLR9 (CD289)	25 leucine-rich repeats. Detailed structure not yet described	Unmethylated CpG DNA	IL-10, pro-inflammatory cytokine production including IL-12p40 production by murine DCs	*C. albicans, A. fumigatus, Cryptococcus neoformans*	(120–123)
Dectin-1 (CLEC7A)	Single extracellular C-type lectin-like domain (CTLD), intracellular immunoreceptor tyrosine-based activation motif	β(1,3)- and β(1,6)-linked glucans	Phagocytosis, cell maturation, respiratory burst, cytokine/chemokine production, direction of T_H_ cell differentiation, CTL priming	*A. fumigatus, C. neoformans, C. albicans, Pneumocystis*	(126, 127, 129, 130, 136–139, 141, 142)
Dectin-2 (CLEC6A)	Single extracellular CTLD, short cytoplasmic tail (couples with FcRγ)	a-mannose structures	Cytokine production, T_H_1 and T_H_17 differentiation	*A. fumigatus, C. albicans, C. neoformans*	(144–147, 150)
Dectin-3 (CLEC4D, CLECSF8)	Single extracellular CTLD, short cytoplasmic tail (does not couple with FcRγ)	a-mannose structures	Phagocytosis, cytokine production, respiratory burst (macrophages)	*C. albicans, C. neoformans* (conflicting studies)	(151, 152, 155, 156, 163)
Mannose receptor (MR, CD206)	Extracellular CR domain, fibronectin type II domain, and 8 CTLDs. Short cytoplasmic tail	Carbohydrates that terminate in d-mannose, l-fucose, or *N*-acetyl glucosamine	Phagocytosis (possibly requires TLR2), fungal clearance, IL-8 production	*A. fumigatus, C. albicans, C. neoformans, Pneumocystis*	(155, 157, 158, 163–166, 168, 170)

*Listed are receptors that have been shown to have a role in fungal infection and are expressed on pDCs. Each receptor is then summarized with a brief description of their structures, their fungal ligands, the major downstream cellular functions, and a selection of sources from which this information was gathered. TLR9, Dectin-1, Dectin-2, Dectin-3, and MR have been implicated specifically in pDC–fungal interaction, while TLR4 has been investigated on other cell types but not yet on pDCs*.

## pDCs in Antifungal Immunity

The involvement of pDCs in antifungal immunity is a topic of study currently emerging in the literature. An early indication came in 2004, when Romani et al. described how thymosin alpha 1, a peptide produced by the thymus, served to activate human cDCs and pDCs by enhancing their phagocytic capacity and IL-10 production in response to *A. fumigatus* stimulation (171). Later, Dan et al. found that mannoproteins from *C. neoformans* and CpG-containing DNA synergistically induced pDCs to produce greater amounts of pro-inflammatory cytokines, although stimulation with mannoproteins alone did not induce any cytokine production (170). This implied that pDCs were able to sense mannoproteins, which are classically recognized by CLRs, and that recognition of mannoproteins acted as an adjuvant for TLR9-dependent pDC activation by CpG. That same year, Ramirez-Ortiz et al. found that *A. fumigatus* DNA contained unmethylated CpG motifs, which triggered TLR9 in mouse and human pDCs and induced cytokine production *in vitro* (122). Perruccio et al. then confirmed that *A. fumigatus* DNA stimulated TLR-9 in pDCs and induced the production of IL-12p70, IL-10, and IFN-α (172).

**Figure 1:**
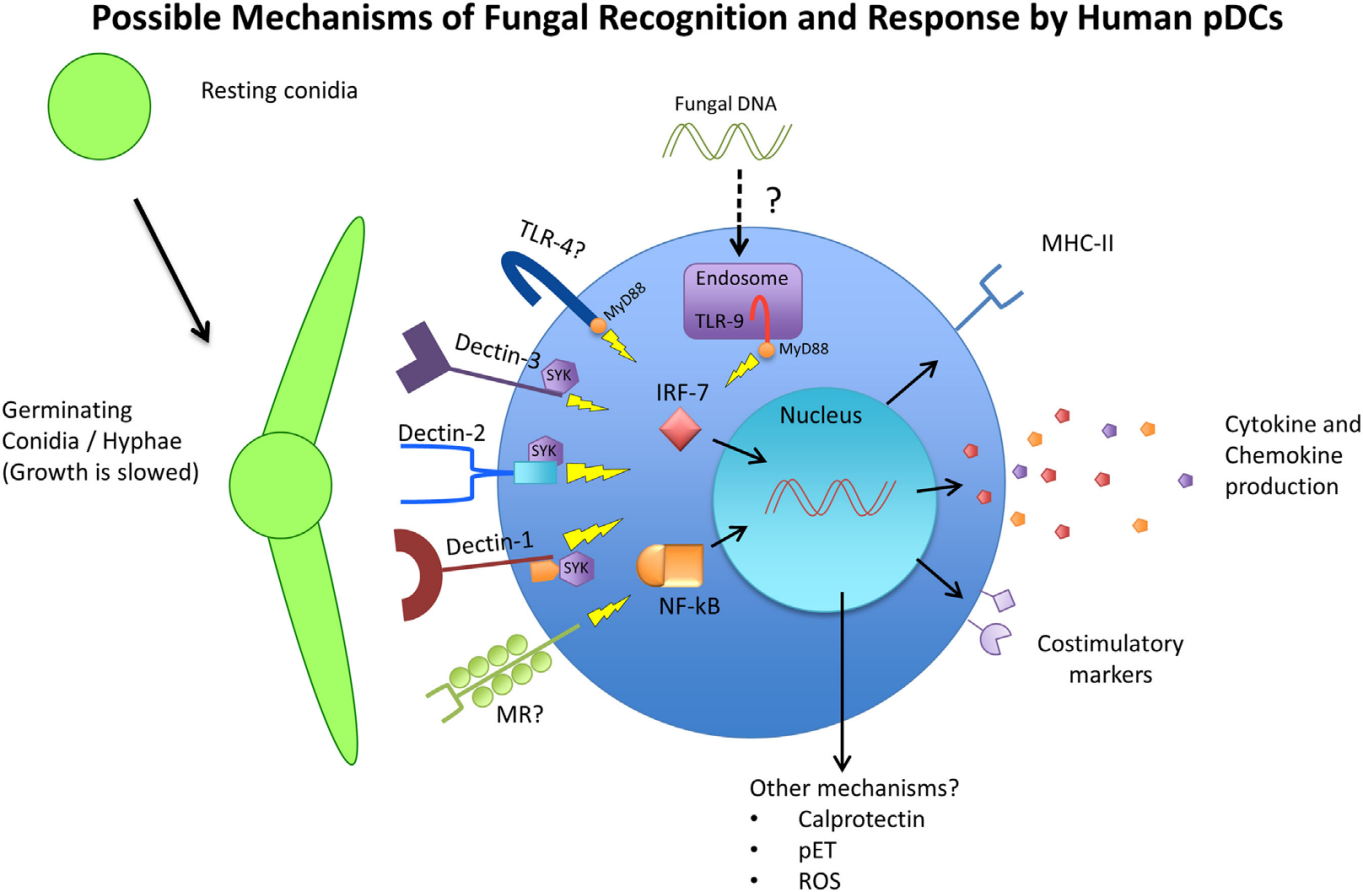
Schematic representing the available receptors and cellular responses of plasmacytoid dendritic cells (pDCs) to fungal pathogens. Resting conidia (RC) or yeast cells are less immunogenic than germinating and hyphal forms of fungi due to the increased availability of ligands such as beta-glucans and alpha-mannan structures of the cell wall. The receptors TLR4, TLR9, Dectin-1, Dectin-2, Dectin-3, and MR have been implicated in antifungal immunity in various models and studies and have also been observed to be expressed by pDCs. The cellular responses of pDCs to fungal stimulation have only begun to be characterized, but include phagocytosis, activation/maturation, cytokine and chemokine production, and MHC-II upregulation. Other pDC antifungal mechanisms that have been proposed include the release of calprotectin by apoptotic pDCs, the production of pDC extracellular traps (pETs), and the production of reactive oxygen species (ROS).

The response of pDCs to fungal recognition is still under investigation, but a few studies have shed some light on mechanisms of pDC antifungal activity (summarized in Figure 1). Direct pDC antifungal activity was first demonstrated in 2011, when Ramirez-Ortiz et al. found that human pDCs directly inhibited growth of *A. fumigatus* hyphae and produced IFN-α and TNF-α in response to *in vitro* hyphal stimulation (173). They found that pDCs spread out over the surface of hyphae and inhibited hyphal viability by up to 80%. However, this strong antifungal activity was observed with a pDC:fungal ratio of 50:1, which seems highly unlikely to occur in a live organism. At a pDC-fungal ratio of 1:10, a 40% decrease in hyphal viability was still observed. At the same time, they found fungal stimulation lead to a large increase in pDC death, mediated partially by the production of gliotoxin by *A. fumigatus*. They also discovered that pDC lysates had the same antifungal activity as live cells, and implicated the release of calprotectin from dying pDCs as a contributor to the inhibition of fungal growth. Calprotectin has a documented role in antifungal immunity as a component of neutrophil extracellular traps (174). However, this was the first indication that pDCs may also use calprotectin. A later study by the same group indicated that pDC-*Aspergillus* interaction seemed to induce the production of pDC extracellular traps (pETs), similar to those extracellular traps laid by neutrophils (175). The production of extracellular traps by pDCs is a novel concept that has not been described elsewhere, and requires careful follow-up. Transcriptome analysis after *A. fumigatus* stimulation revealed the upregulation of several genes involved in activation, chemokine production, and antigen presentation, as well as genes associated with apoptosis. *In vivo*, mice depleted of pDCs were significantly more susceptible to *Aspergillus-*induced mortality, both after pulmonary and intravenous challenge (173). In addition, pulmonary challenge with *A. fumigatus* caused a six-fold increase in pDC number in the lungs. In a *C. neoformans* model, Hole et al. indicated that murine and human pDCs could phagocytose yeast cells and could directly inhibit *C. neoformans* growth through the use of reactive oxygen species (72).

As mentioned above, pDCs express receptors that are known to be involved in fungal recognition (summarized on Figure 1). As early as 2002, Taylor et al. mentioned that murine pDCs express dectin-1, although the data were not shown. Seeds et al. (71) later confirmed that finding, and added that murine pDCs also expressed the CLRs dectin-2 and MR (71). Because murine pDCs are not perfect representations of human pDCs, these receptors also needed to be investigated on human pDCs. Studies on human pDCs observed the expression of dectin-1 (176), dectin-2 (177), and MR (70, 178). However, attempts to identify which pDC receptor is responsible for fungal recognition have delivered mixed results. One study singled out dectin-2 as the main receptor responsible for recognition of *A. fumigatus* hyphae by human pDCs (175). They used laminarin and mannan to block dectin-1 and dectin-2, respectively, and found that while both blocking agents decreased the ability of pDCs to associate with fungal hyphae, only mannan’s effect reached statistical significance. However, the definition and use of mannan as a dectin-2 blocker may be problematic, as it can be the ligand for other CLRs, including dectin-3 and MR. The study did follow-up by blocking with specific antibodies against dectin-1 and dectin-2, which recapitulated their results that dectin-2 was more responsible for pDC–hyphal association. Hole et al. observed the presence of dectin-3 on murine pDCs, and demonstrated that blocking dectin-3 on human pDCs resulted in a blunted reaction to *C. neoformans* stimulation (72). In their study, pDCs from dectin-3 knockout mice experienced the greatest reduction in fungal uptake and growth inhibition, greater than dectin-1, dectin-2, and MR knockout mice. Accordingly, pDCs from dectin-3 knockout mice were greatly deficient in their ability to phagocytose *C. neoformans* yeast cells. It is interesting to note that these findings stand in contrast to the aforementioned study where dectin-3 was not required for protection against *C. neoformans* (156). Nevertheless, the data again indicated that while dectin-3 knockout mice were the most affected in their antifungal activity and the only ones to achieve significance, knocking out dectin-1, dectin-2, and MR also resulted in varying degrees of reduced antifungal activity. This allows the possibility that pDCs use a combination of receptors to recognize and respond to fungal pathogens and that these PRRs have a measure of redundancy.

Considered together, this evidence strongly indicates that pDCs play an important role in antifungal immunity. However, there remains much to be discovered concerning the mechanisms of pDC–fungal interaction, cellular responses to recognition, and the role of pDCs in the broader landscape of antifungal immunity.

## Concluding Remarks

One of the most insidious threats to the health and well-being of immunocompromised individuals, including those living with HIV, is the threat of opportunistic infections. Opportunistic fungal infections can be particularly fast, lethal, and difficult to treat. The main contributing factor for increased susceptibility to fungal infection in immunocompromised patients is the depletion and dysregulation of many components of the immune system; even when infection is well-controlled with antiretroviral medication, over time this dysregulation is measurable in individuals chronically infected with HIV. The pDC is no exception; HIV patients have a decline in number and functionality of their pDC compartment. This is especially problematic when considering the role of pDCs in fungal infection that is currently emerging in the literature. Studies have shown that pDCs have the machinery to recognize and respond to fungal infection, and do so in a manner that indicates pDCs are involved in a successful antifungal immune response. Therefore, dysregulation of the pDC compartment may be a contributing factor to the increased susceptibility of fungal infections that afflict HIV patients and other immunocompromised populations.

## Author Contributions

SM performed all literature searches and writing of this review. PF-B provided focused expertise and guidance on the field of plasmacytoid dendritic cell and HIV biology, extensive revision of this manuscript, and final approval of the version for publication.

## Conflict of Interest Statement

The authors declare that the research was conducted in the absence of any commercial or financial relationships that could be construed as a potential conflict of interest.
